# Thermal Conductivity of Silver Nanowire–Polymer Composites Prepared via Layered Assembly

**DOI:** 10.1021/acsapm.4c03095

**Published:** 2025-01-29

**Authors:** Matthew L. Fitzgerald, Zhiliang Pan, Godfrey Sauti, Deyu Li

**Affiliations:** † Department of Mechanical Engineering, Vanderbilt University, Nashville, Tennessee 37235, United States; ‡ NASA Langley Research Center, Hampton, Virginia 23681-2199, United States

**Keywords:** nanocomposites, thermal boundary resistance, thermal conductivity, silver nanowires, polymer composites

## Abstract

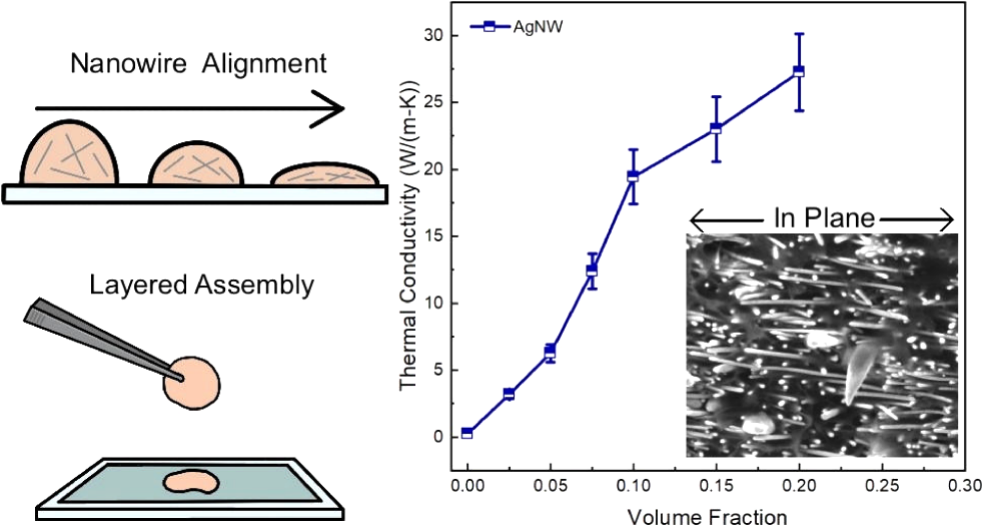

Thermally conductive polymer composites are of great interest for a variety of applications. One strategy to enhance the composite thermal conductivity is to minimize the thermal resistance at numerous contacts and interfaces inside the composites. Recently, it has been shown that the thermal boundary resistance between silver nanowires (AgNWs) and polyvinylpyrrolidone (PVP) is significantly lower than that between nonmetallic nanofillers such as carbon nanotubes and various polymers. To demonstrate that AgNWs could serve as effective fillers for thermally conductive composites, here we report on preparation and characterization of AgNW-PVP composite thin films. A layered assembly technique, which allows for the embedded filler network to largely align along the in-plane direction, has been adopted to prepare composite films of various AgNW volume fractions. Thermal measurements show that the combined effects of aligned AgNWs and low AgNW-PVP interfacial thermal resistance lead to remarkably enhanced in-plane thermal conductivity. At an AgNW volume fraction of 0.2, the composite thermal conductivity reaches 27.2 W/(m·K), which represents more than 2 orders of magnitude enhancement as compared to that of the corresponding neat polymer. Importantly, analyses disclose a nonmonotonic trend for the effective thermal conductivity of the AgNW network, which could be due to the more significant contact resistance at a higher AgNW loading level. This study provides insights into manufacturing highly thermally conductive polymer composites for thermal management applications.

## 1 Introduction

Polymers are a unique and versatile class of materials categorized by their low cost, wide availability, and desirable mechanical properties, which make them ideal structural materials in a broad range of applications. A long-standing issue for most polymers, however, is the poor thermal conductivity in their bulk forms. While it has been shown that the covalently bonded individual polymer chains could have very high thermal conductivity along the chain,^1,2^ bulk polymers composed of a random network of entangled polymer chains with weak interchain van der Waals interactions usually have thermal conductivities on the order of 0.1 W/(m·K).^3^ This ultralow thermal conductivity of bulk polymers significantly limits their applications in systems where effective heat dissipation is critical for device performance, such as flexible electronics,^4^ photovoltaic cells,^5^ and thermal interface materials.^6^ As a result, considerable efforts have been put forth seeking to enhance the thermal properties of bulk polymers, often by introducing high thermal conductivity fillers into the polymer matrix.

Notably, metallic nanofillers have demonstrated considerable promise for thermally conductive polymer composites. For example, Huang et al. reported a peak thermal conductivity of 6.5 W/(m·K) at a volume fraction of 0.2 for silver nanoparticle (AgNP)-poly(vinylidene fluoride) (PVDF) composites.^7^ A more impressive result from Balachander et al. suggested a thermal conductivity of ∼5.5 W/(m·K) at a loading level of merely 0.03 for gold nanowire (AuNW)-polydimethylsiloxane (PDMS) composites.^8^ More recently, Chen et al. measured the thermal conductivity of copper nanowire (CuNW) filled epoxy and graphite filled epoxy and found that at a low loading level of only 0.12 vol %, the CuNW-epoxy composite had a thermal conductivity of 2.59 W/(m·K), which was higher than the measured value (0.86 W/(m·K)) for the graphite-epoxy composite at a loading level of 1 vol %.^9^ Recently, the thermal boundary resistance between individual AgNWs and PVP was measured to understand the better performance of metallic nanofillers. The results indicated that the thermal boundary resistance at Ag-PVP interfaces could be 1 order of magnitude lower than the corresponding values between carbon nanotubes (CNTs) and various polymers.^10^ Together, these results suggest that even though coupling between electrons and phonons must be involved at metal–polymer interfaces, metallic fillers could serve as more effective filler materials for thermally conductive composites.

To further explore the effect of the low Ag-PVP interfacial thermal resistance on the composite thermal conductivity, we prepared AgNW-PVP composite films through a layered assembly approach and measured the thermal conductivity of the resulting films. Here, individual composite layers with a thickness of ∼10 μm were cast from a dilute suspension of PVP and AgNWs in ethanol, which could be “welded” together through a wetting and stacking process. This fabrication technique allows for the embedded nanowires to largely align to the in-plane direction as the length of the nanowires (40 μm) is significantly larger than the individual layer thickness. The alignment and the low thermal boundary resistance between AgNWs and PVP^10^ lead to a measured in-plane thermal conductivity up to 27.2 W/(m·K).

## 2 Experimental Section

### 2.1 Composite Fabrication

**Figure 1:**
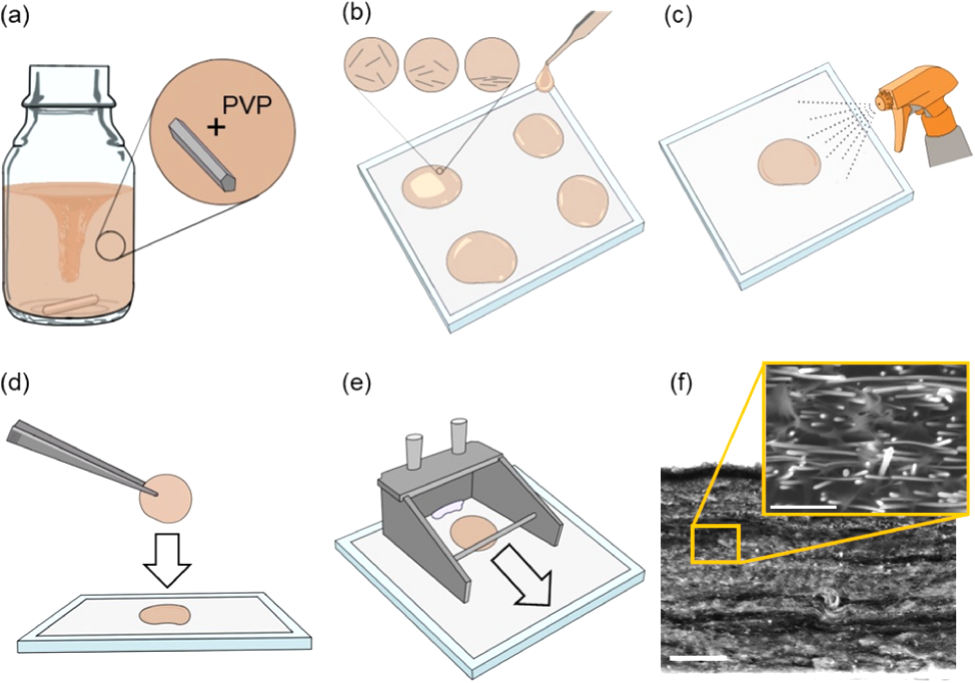
Schematic diagram of the layered assembly process. (a) PVP powder is added to the AgNW/Ethanol solution and magnetically stirred overnight. (b) A glass pipet is used to cast an array of ∼40 mm diameter droplets onto a glass plate mounted release liner. AgNWs settle to the in-plane direction during the ethanol evaporation process. (c) A single layer is isolated, and a mister spray bottle is used to gently coat and partially dissolve the upper surface of the composite layer. (d) Tweezers are used to manually stack a cured layer onto the partially dissolved surface of the base layer to “weld” the layers together. Steps (c)–(d) are then repeated as necessary to achieve the desired composite film thickness. (e) A doctor blade is used to coat the as-prepared film with a layer of neat PVP. The PVP serves as an insulation layer and is required for the thermal measurements. (f) An SEM micrograph of an assembled PVP-AgNW thin film. The scale bar is 25 μm. The inset SEM micrograph shows a high-magnification view of the assembled composite demonstrating that the embedded AgNWs are largely aligned to the in-plane (horizontal) direction. The inset scale bar is 1 μm.

The schematics in Figure 1a–e illustrate the layered assembly procedure. In order to prepare the composite solution, PVP powder (Sigma-Aldrich, 437190-25G, MW = 1,300 kg/mol) was dissolved directly into the as-received solution of AgNWs suspended in ethanol (Sigma-Aldrich, 807923-25 ML, specified AgNW dimension: diameter × length: 70 nm × 40 μm). The required amount of PVP powder for a given volume fraction is calculated by , where *m*_*PVP*_ is the required mass of PVP to be added to the solution, *m*_*Ag*_ is the mass of silver in the nanowire suspension, *ρ*_*Ag*_ is the density of silver with a known value of 10.49 g/cm^3^, φ is the desired volume fraction of AgNWs in the composite, and *ρ*_*PVP*_ is the density of PVP taken as 1.25 g/cm^3^.^11^ The nanowire suspensions from Sigma-Aldrich have an AgNW concentration of 5 mg/mL and a total volume of 25 mL. Consequently, the total mass of silver in each vial is 0.125 g, and the required mass of PVP for each volume fraction can be readily calculated. Note that it has been previously demonstrated that adequate in-solution mixing is critical to achieve a homogeneous filler dispersion in the cured composite.^12^ As such, the solution was magnetically stirred for ∼16 h before the thin film layer casting (Figure 1a).

A section of fluorinated release liner was affixed to a flat glass plate resting on a leveled surface prior to drop-casting. The release liner was necessary for the composite film preparation as it was discovered that individual composite layers would not release from a bare glass plate without rupturing. In contrast, when using the release liner composite layers as thin as 5 μm could be easily peeled off from the liner surface with a pair of tweezers. A small amount of the composite solution was drawn into a glass pipet once a homogeneous dispersion of PVP in the AgNW-ethanol mixture was prepared, and then a series of ∼40 mm diameter droplets were cast onto the surface of the release liner (Figure 1b). Subsequently, the droplets were left in ambient conditions for 1–2 h until the ethanol completely evaporated, leaving behind individual PVP thin film layers with AgNW fillers.

Once individual AgNW-PVP composite film layers cast on the release liner had fully cured, a single layer was selected as the base to assemble a thicker composite film, and the remaining films were removed and set aside. In most cases, this film removal could be accomplished by peeling from one edge with a set of sharp tweezers and carefully separating the composite layer from the release liner; however, for thinner films a razor blade could assist in the film separation from the release liner.

For the assembly of multiple layers, a misting spray bottle was used to apply a small amount of ethanol to the surface of the selected base layer. The applied ethanol wet the entire surface without dissolving the film layer (Figure 1c). Immediately following the wetting process another cast layer was manually placed on top of the wetted base layer such that the two layers would be welded together (Figure 1d). Note that during this assembly, the top layer was placed onto the wetted bottom layer with one edge coming into contact first, and then the top layer was gradually “rolled” onto the surface to minimize any trapped air or ethanol. After the top layer was fully laid down, slight physical pressure was applied to any areas where bubbles could be discerned. Once the layers had fully dried, the process was repeated until a desired film thickness had been achieved.

We measured the in-plane thermal conductivity of the resulting composite films using a DC heating method with a thin layer of gold as the resistance heater and thermometer.^13,14^ Since the composite film is electrically conductive, we first coat the AgNW-PVP composite film with a thin layer of electrically insulating neat PVP. To do so, a solution with 20 vol % of PVP in ethanol was prepared. Following the standard doctor blading procedure, a small amount of the solution was poured directly onto the release liner next to the composite film, and an adjustable doctor blade was used to drag the dissolved PVP over the surface of the composite film at the desired thickness.^15^ The resulting insulation layer is 10–20 μm thick and is functionally equivalent to the dielectric layers used to insulate electrically conductive films from resistive heaters.^13^ Importantly, the resulting surface is smooth enough for deposition of 10 nm thick continuous gold film with consistent electrical resistance.

E-beam evaporation was used to deposit a 10 nm thick gold film serving as a resistive heater and thermometer for thermal characterization. The gold film was deposited on the insulated surface of the composite thin films using an Angstrom AMOD Physical Vapor Deposition Platform. Finally, the composite film was cut into ∼0.8 mm wide by ∼30 mm long samples for thermal measurements. A scanning electron microscopy (SEM) cross-sectional view of the composite in which the layered structure can be perceived is shown in Figure 1f.

Fabricating composite materials using the layered approach described above, with individual layers cast from a dilute suspension, allows for a homogeneous structure with the embedded AgNWs largely aligned along the in-plane direction. This is achieved primarily because the length of the AgNWs (40 μm) is significantly greater than the individual composite layer thickness (∼10 μm).

The thickness of the individual composite layers was found to be inversely correlated to the volume fraction of embedded AgNWs. This is due to the lower nonvolatile PVP concentration at higher AgNW volume fraction in the droplets of roughly the same diameter (∼40 mm). The layer thickness was estimated by dividing the total composite sample thickness, as measured by SEM, by the number of assembled layers, which ranged from ∼4 μm for the sample with an AgNW volume fraction of 0.2 to ∼25 μm for those with a AgNW volume fraction of 0.025. Therefore, while the AgNWs were aligned primarily to the in-plane direction across all measured samples, the decreasing layer thickness with increasing AgNW volume fraction would likely result in a greater degree of in-plane alignment (anisotropy) for samples with higher AgNW concentrations.

### 2.2 Thermal Conductivity Measurement

**Figure 2:**
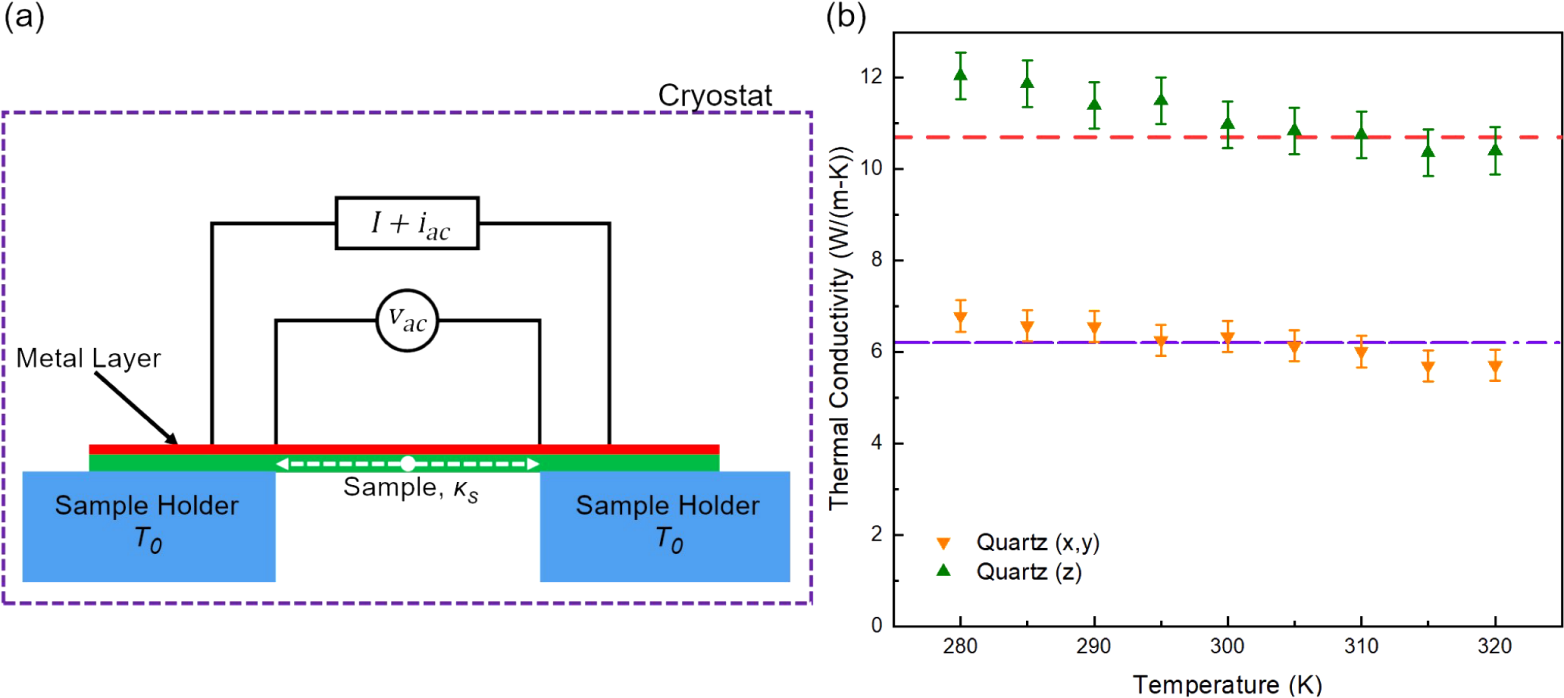
Thermal measurement scheme. (a) Schematic of the thermal circuit for the steady-state DC heating measurement setup. (b) Measured thermal conductivity of quartz along (*x*,*y*) and (*z*) crystalline directions with reported literature values at 300 K (dashed lines).^17−19^

The sample in-plane thermal conductivity was measured using the steady-state direct current (DC) heating approach (shown schematically in Figure 2a), which has been widely implemented to characterize the thermal conductivity of various materials including crystalline silicon thin films,^13^ CNT composite microfibers,^14^ and metallic nanofilms.^16^ Under this measurement scheme, the composite samples with the deposited gold layer are suspended between two heat sinks which here take the form of machined Cu holders with a nominal gap distance of 6.35 mm. The Cu holder with the mounted sample was first placed in a cryostat (Janis, Model CCS-400/204) and maintained at a constant temperature, *T*_*0*_, under a high vacuum condition (<1 × 10^–6^ mbar) to achieve thermal equilibrium. Subsequently, a small alternating current (AC) signal (*i*_*ac*_) was coupled to a DC source by an integrated differential amplifier (Analog Devices SSM2141) and applied to the gold heater layer. The DC current (*I*) generated Joule heat in the metal layer, and the resulting temperature rise induced an electrical resistance change of the Au layer that was measured via a four-probe method by monitoring the corresponding changes in the AC voltage (*v*_*ac*_) across the suspended sample (see additional details in Supporting Information).

Assuming negligible contact thermal resistance between the sample and the Cu holder and insignificant radiation heat losses (see Supporting Information), the temperature profile of the sample can be described based on a one-dimensional (1-D) conduction model such that^16^
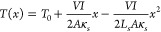
1where *T* is the temperature that follows a parabolic profile along the sample length direction, *V* is the applied DC voltage, *A* is the sample cross-sectional area, *κ*_*s*_ is the effective thermal conductivity of the suspended sample including the composite film and metal layer, *L*_*s*_ is the sample suspended length, and *x* is the distance along the suspended sample with *x*(0) and *x*(*L*_*s*_) representing the edge locations of the suspended segment. To ensure that this simple 1-D model is applicable to the AgNW-PVP sample, we have conducted finite element analysis (FEA) to model the heat transfer process in the composite materials, as discussed later.

Further, the average temperature rise of the film, , can be described according to eq. 2. Note that the heating power (*VI*) is selected such that the maximum temperature rise is small (<5 K).^16^
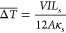
2

The measurement was conducted by applying a sweeping DC current at each measurement temperature and recording the corresponding change in resistance (*R*). From the resulting *R-I* profile, the resistance at each base temperature with zero applied heating power (*R*_*0*_) and the temperature coefficient of resistance (TCR) of the Au layer can be readily obtained (see Supporting Information). Therefore, it can be shown that the sample thermal conductivity (*κ*_*S*_) is related to the resistance change of the metal layer according to^16^
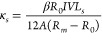
3where β is the TCR and *R*_*m*_ is the measured electrical resistance at a given applied heating power.

Note that the thermal conductivity given by eq. 3 is the effective thermal conductivity of the measured samples including the 10 nm gold layer, the PVP insulation layer, and the AgNW-PVP composite layer such that
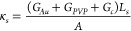
4where *G*_*Au*_, *G*_*PVP*_, and *G*_c_ are the thermal conductance of the Au, PVP, and the AgNW-PVP composite layers, respectively. It can be shown from Weidemann-Franz law that *G*_*Au*_ = *LT*/*R*, where *L* is the Lorenz number and *R* is the electrical resistance. The Lorenz number can be taken as the Sommerfeld value of 2.44 × 10^–8^ (W·Ω)/K^2^ for approximate estimation. The resistance of the gold layer was determined to be ∼200 Ω for the current samples (see Supporting Information). Thus, the value of *G*_*Au*_ is estimated as 3.66 × 10^–8^ W/K, which accounts for just 0.02% of the total sample thermal conductance in the validated temperature range of the steady-state DC measurement technique. As such, the thermal conductance of the deposited Au layer is considered negligible.

Similarly, the thermal conductivity of neat PVP has been measured to be 0.23 W/(m·K),^10^ which corresponds to a thermal conductance of 5.07 × 10^–7^ W/K for a 20 μm thick PVP insulation layer or less than 0.28% of the total sample thermal conductance. Thus, the thermal conductance of the PVP insulation layer can also be considered negligible. Therefore, eq 3 reduces to *κ*_*s*_ = *G*_*c*_*L*_*s*_/*A*_*c*_, where *A*_*c*_ denotes the cross-sectional area of the AgNW-PVP composite. Note that the thickness of the neat PVP insulation layer can be easily extracted from SEM micrograph of the samples.

To validate the measurement approach, single crystalline quartz samples with well-documented thermal conductivity values along different crystalline directions were first measured. Samples were derived from 200 μm *x*-cut (Precision Micro Optics, PSQB-13D332) and *z*-cut (Precision Micro Optics, PSQB-33D232) quartz wafers. Following the procedure outlined for the composite samples, the wafers were diced into 0.85 mm wide bars, and 10 nm of Au was deposited on one surface to serve as the heater and thermometer.

The measured thermal conductivities along the (*x*,*y*) and (*z*) crystalline directions are presented in Figure 2b, which are in good agreement with the values reported at 300 K of 6.6 W/(m·K) for the (*x*,*y*) directions and 11.0 W/(m·K) for the (*z*) direction.^17−19^ While this is a relatively narrow range of validated thermal conductivities, the steady-state DC heating technique measures the sample total thermal conductance, *G*. Thus, samples of higher or lower thermal conductivity can be measured, so long as the sample dimensions are such that the total thermal conductance falls within the validated range. Note that it is also important to ensure that the radiation heat loss from the sample surface is negligible as compared to the sample conductance.

## 3 Results and Discussion

**Figure 3:**
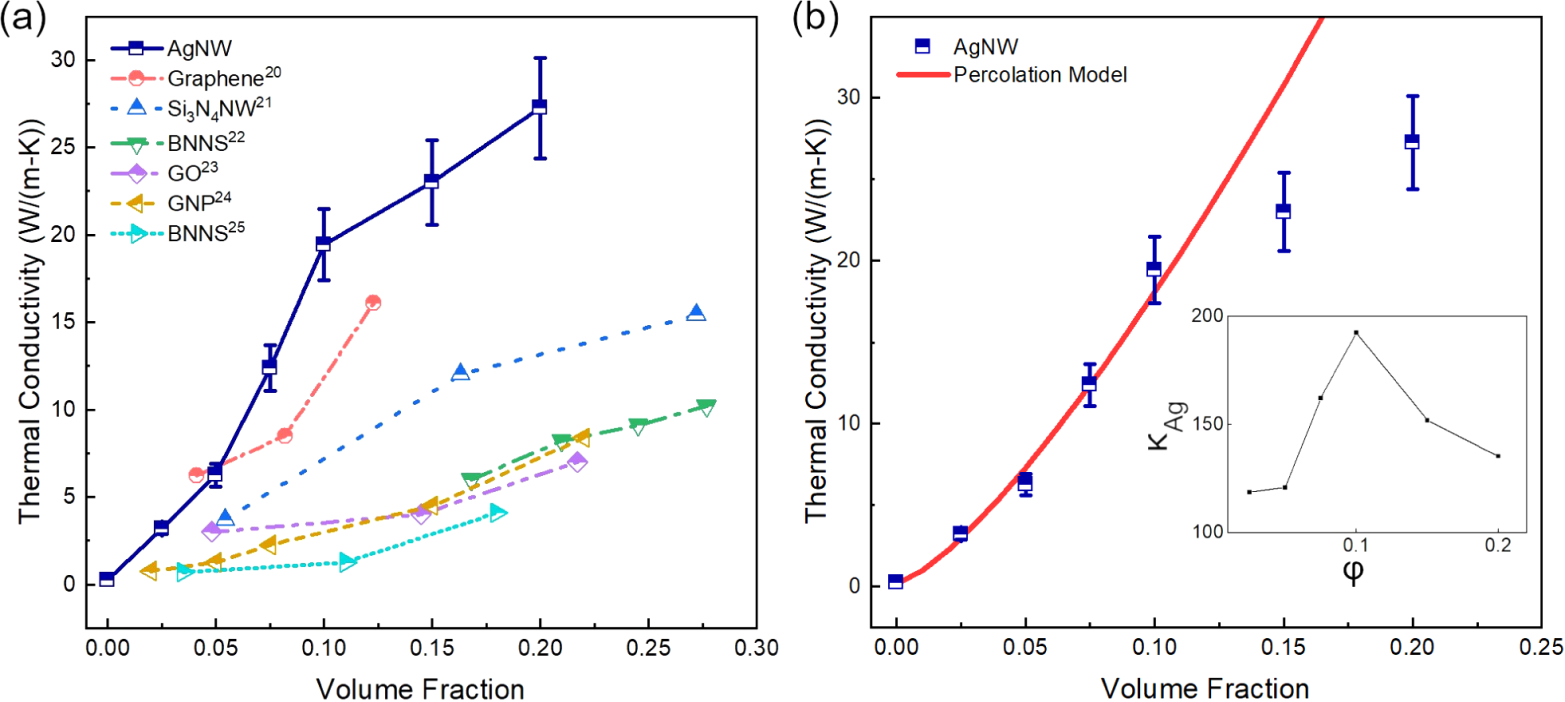
Thermal measurement results. (a) Measured in-plane thermal conductivity of the AgNW-PVP composite samples as compared to in-plane thermal conductivity values reported recently for composite samples with various fillers. (b) Fitting results for φ ≤ 0.1. The inset shows the effective thermal conductivity of the percolated AgNW network. The estimation of the uncertainty can be found in Supporting Information.

AgNW-PVP composite film samples with a range of AgNW volume fractions including 0.025, 0.05, 0.075, 0.1, 0.15, and 0.2 were fabricated and measured. Three different samples were measured for each AgNW volume fraction except the highest one, for which five samples were measured. The extracted in-plane thermal conductivity versus the AgNW volume fraction is plotted in Figure 3a. Notably, even at the lowest AgNW volume fraction of 0.025, the composite samples demonstrate a thermal conductivity of 3.2 W/(m·K), which is more than 1 order of magnitude higher than that of the neat PVP. The measured thermal conductivity increases with the AgNW volume fraction, reaching 27.2 W/(m·K) when the AgNW volume fraction reaches 0.2. This represents over 2 orders of magnitude thermal conductivity enhancement relative to the value of neat PVP polymer.

To compare the effectiveness of different nanofillers in enhancing the composite thermal conductivity, we also plot the reported in-plane thermal conductivity of composites with various other nanofillers^20−25^ together with the AgNW-PVP composites. Interestingly, the AgNW-PVP composites demonstrate a consistently higher in-plane thermal conductivity among all surveyed studies, despite the lower thermal conductivity of AgNWs than those for graphene^26^ and boron nitride.^27^ One important contributing factor for this could be the lower thermal boundary resistance between silver and PVP as compared to that between other stiffer nanofillers and the polymer matrix.^10^

Different models based on the effective media theory have been developed to predict the thermal conductivity of polymer composites. Through a trial and error process, we selected a nonlinear percolation model proposed by Foygel et al.^28^ to fit the experimental data to further understand the effect of AgNW volume fraction on the measured thermal conductivity. In this model, the thermal conductivity of a composite material is calculated as
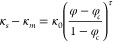
5where *κ*_*m*_ is the thermal conductivity of the matrix material, *κ*_*0*_ is a prefactor that depends on the thermal conductivity of individual nanofillers, the contact thermal resistance between nanofillers, and the topology of the percolated network, φ is the filler volume fraction, *φ*_*c*_ is the critical volume fraction or the percolation threshold, and τ is an exponent that is dependent on the filler aspect ratio, *p*. For the AgNW-PVP composites, the aspect ratio is defined as the nanowire length, *l*, divided by the diameter, *d*, (*p = l/d*). For high aspect ratio fillers (*p* ≫ 1), the critical volume fraction is related to the aspect ratio such that *φ*_*c*_ ≈ 0.6/*p*.^28^ In particular, for the AgNWs used in this study, the aspect ratio is 571, and the critical volume fraction is 0.001. The remaining unknowns in eq. 5 are *κ*_*0*_ and τ, which are taken as fitting parameters.

Interestingly, an excellent fit of the experimental data could be achieved when excluding the measured thermal conductivity values for the samples with φ > 0.1. It should be noted that even though the AgNWs are aligned to a greater degree as the AgNW volume fraction increases, the composite thermal conductivity for φ > 0.1 falls below the model prediction based on the best fitting to the data for φ ≤ 0.1.

In order to better understand this result, a simple parallel conduction model was employed to calculate the effective thermal conductivity of the percolated AgNW network for each volume fraction such that
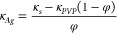
6where *κ*_*Ag*_ is the effective thermal conductivity of the AgNW network and *κ*_*PVP*_ is the thermal conductivity of PVP. Equation 6 reveals that the thermal conductivity of the AgNW network achieved a peak value of 192 W/(m·K) at a volume fraction of 0.1 and then actually began to decrease as the AgNW volume fraction further increases, as shown in the inset of Figure 3b. One possible explanation for this unexpected trend is that the number of filler–filler contacts will necessarily increase as the filler volume fraction escalates. At lower AgNW concentrations, increasing the filler volume fraction has the potential to bridge gaps between fillers and increase the total number of percolated heat transfer paths for an unsaturated composite, i.e., one which contains some incomplete thermal pathways along the heat transfer direction. However, once the AgNW volume fraction exceeds 0.1, most AgNWs are part of percolated networks. In this case, the benefit of adding additional AgNWs is reduced as the AgNW network would have more filler–filler contacts, which leads to a lower effective thermal conductivity of the AgNW network. Indeed, the contact thermal resistance between individual AgNWs with a thin PVP interlayer was previously determined to be ∼10^7^ K/W, which is of the same order of magnitude as the total thermal resistance along the length of individual AgNWs used in this study.^10^ It is important to note that the overall thermal conductance of the AgNW network keeps increasing with the AgNW volume fraction; however, as the benefit of additional AgNWs becomes less significant for φ > 0.1, the effective thermal conductivity of the AgNW network, which is normalized with the volume fraction, could decrease according to eq. 6.

Using eq. 5, the best fit of the experimental data with φ ≤ 0.1 was achieved with *κ*_*0*_ = 377 ± 198 W/(m·K) and τ = 1.32 ± 0.14, where the given uncertainty represents the 95% confidence interval (see Supporting Information), and fitting results are plotted alongside the measured composite thermal conductivity in Figure 3b. Importantly, Foygel et al. also showed that the total interfacial thermal resistance, *R*_*i*_, is related to the fitting parameters *κ*_*0*_ and τ, the critical volume fraction, and the length of the filler particles, *l*_*Ag*_, as given by eq. 7.^28^
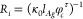
7

The resulting value of *R*_*i*_ is (6.01 ± 4.39) × 10^5^ K/W.

Finally, the average overlap area of two adjacent nanowires, , is calculated according to eq. 8.^29,30^
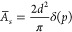
8where
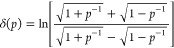
9

The contact area determined from eqs. 8 and 9 is 2.2 × 10^–14^ m^2^ which, when combined with the total thermal resistance calculated from eq. 7, yields an interfacial thermal resistance between AgNW and PVP for unit area of (1.32 ± 0.96) × 10^–8^ m^2^·K/W. This interfacial thermal resistance is about twice of the previous result of (5.5 ± 4.86) × 10^–9^ m^2^·K/W for the corresponding value measured at the individual contact level.^10^

As noted previously, the measurement scheme assumes 1-D heat conduction along the ribbon length direction, which has been widely adopted in this type of measurements. This assumption is valid so long as the thermal resistance in the thickness direction is much lower than that along the in-plane direction.^13^ In this measurement, while the sample thickness (up to 200 μm) is much smaller than the suspended sample length (6.35 mm), the cross-plane thermal conductivity, especially that of the ∼20 μm thick neat PVP insulation layer, is much smaller than the in-plane thermal conductivity of the composite. Therefore, a finite element analysis (FEA) was conducted to determine the experimental error introduced by adopting the 1-D conduction model, even though estimates reveal that the thermal resistance in the thickness direction is still significantly lower than that in the length direction.

**Figure 4:**
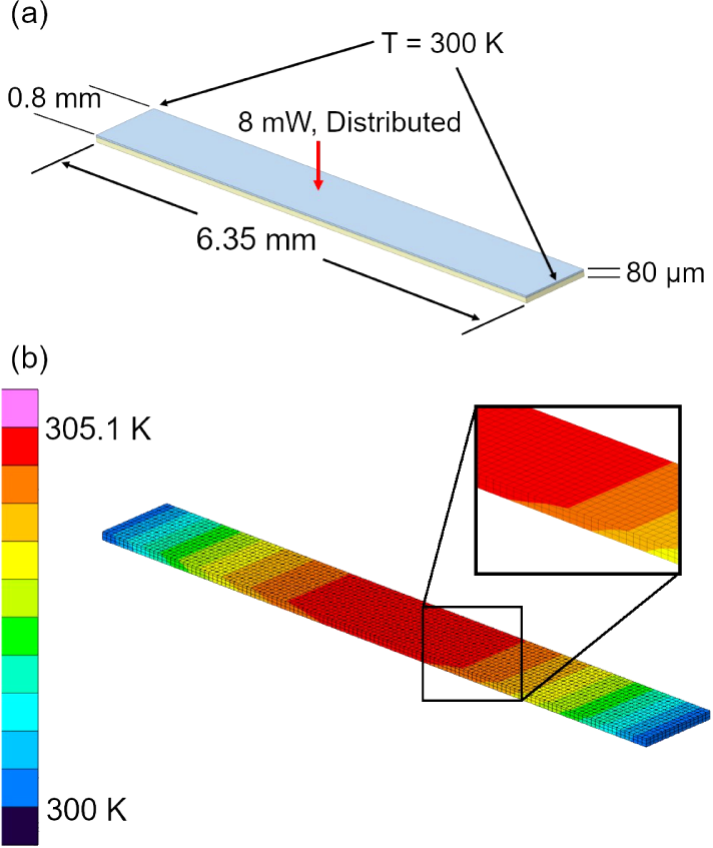
CAD and FEA modeling and results. (a) CAD model geometry with PVP shown in blue and the composite sample shown in yellow. Upon import into Thermal Desktop and 8 mW distributed heat load is applied to the upper surface and a fixed boundary temperature of 300 K is applied to the ends of the sample. (b) Steady-state FEA temperature distribution in Kelvin. The inset shows the thermal gradient through the sample thickness.

A computer-aided design (CAD) model of the suspended portion of the measured samples was assembled in SpaceClaim and imported into Thermal Desktop, a commercially available thermal modeling software. The CAD model dimensions were selected to be representative of the measured composite samples with an AgNW volume fraction of 0.2, and the model has a length, width, and thickness of 6.35 mm, 0.8 mm, and 80 μm, respectively, which are consistent with the sample dimensions in the experimental studies. Of the 80 μm thickness, the lower 60 μm was modeled as a homogeneous structure with a thermal conductivity of 27.2 W/(m·K) along the in-plane and 0.23 W/(m·K) along the cross-plane directions. This corresponds to a worst-case scenario consideration because even though the AgNWs are largely aligned along the in-plane direction, the cross plane thermal conductivity should still be much higher than 0.23 W/(m·K). The upper 20 μm is modeled as neat PVP with an isotropic thermal conductivity of 0.23 W/(m·K). The boundary conditions applied to the thermal model are the same as those used in the derivation of the 1-D conduction model given by eq. 1 and consist of a fixed boundary temperature (300 K) at the two ends of the suspended section, and a distributed heat load applied (8 mW) to the top surface of the PVP insulation layer. A schematic of the CAD model and the thermal model boundary conditions is provided in Figure 4a.

The thermal gradient for the steady-state solution of the FEA model is shown in Figure 4b. In a purely 1-D conduction condition, each cross-section along the suspended sample would have a uniform temperature such that the average temperature of the sample is equal to the average temperature of the upper surface as measured by the 10 nm gold heater-thermometer layer. However, a lower cross-plane thermal conductivity results in a thermal gradient along the thickness of the suspended sample (Figure 4b inset) and a higher average temperature as measured by the deposited gold layer. As a result, only the upper surface of the PVP insulation layer was considered when calculating the average temperature rise of the FEA model.

The average temperature rise of the upper surface of the FEA model was determined to be 3.47 K, which is 6.4% higher than the 3.26 K calculated from the idealized 1-D conduction model (eq. 2). If the FEA model is modified and the PVP insulation layer removed, it can be shown that the difference is reduced to 2.4%. This suggests that a 4% increase in the average temperature from the ideal model can be attributed to the PVP insulation layer. Importantly, the above analysis indicates that a maximum of ∼6% error will be introduced for the worst-case scenario, since the measured thermal conductivity is inversely proportional to the average temperature rise.

## 4 Conclusion

In summary, a facile layered fabrication technique was employed to assemble AgNW-PVP composites with significantly enhanced thermal conductivity along the in-plane direction. Measurements based on the steady-state DC thermal bridge method demonstrate monotonic thermal conductivity enhancement as the AgNW volume fraction escalates, with a maximum value of 27.2 W/(m·K) at an AgNW volume fraction of 0.2, over 2 orders of magnitude higher than the corresponding value of neat PVP. These data further confirm the understanding gained through studies at individual nanostructure level and provide insights into manufacturing thermally conductive polymer composites.

## Supporting Information Available

Sample preparation, measurement method, contact thermal resistance characterization, radiation loss characterization, measurement method validation, cross-sectional area characterization, and experimental uncertainty (PDF)
