# Supplementary material for: Thermal Conductivity of Silver Nanowire–Polymer Composites Prepared via Layered Assembly
Source: ACS Appl Polym Mater. 2025 Jan 29;7(3):1394–400. doi: 10.1021/acsapm.4c03095 (PMC11833763; doi:10.1021/acsapm.4c03095)
Supplement: Supplementary file 1 — ap4c03095_si_001.pdf [file ap4c03095_si_001.pdf]

Supporting Information

for

Thermal Conductivity of Silver Nanowire-Polymer Composites Prepared via Layered Assembly

Matthew L. Fitzgerald,[1,*] Zhiliang Pan,[1] Godfrey Sauti,[2] and Deyu Li[1,*]

[1]Department of Mechanical Engineering, Vanderbilt University, Nashville, TN, 37235, USA
[2]NASA Langley Research Center, Hampton, VA 23681-2199, USA

[*]Corresponding authors: matthew.f@wustl.edu, deyu.li@vanderbilt.edu

Contents

I. Experimental Setup and Procedure 3

II. Measurement Method 4

III. Contact Thermal Resistance 5

IV. SEM Images of the Sample Cross-Sections..6

V. Effects of Thermal Radiation... 6

VI. Measurement Uncertainty 7

VII. Modelling Uncertainty 9

I. Experimental Setup and Procedure

After preparing the samples as outlined in the main text, a commercially available, electrically insulating thermal paste with a reported thermal conductivity of 15.7 W/(m-K) (Shenzhen Tensan Co., Ltd, SYY-157) was applied to the copper sample holder, and the samples were pressed firmly into the holder surface. Electrical connections were then established by using silver paint (Ted Pella, INC., 16040-30) to attach flat contact pads to the gold coated sample surfaces. The contact pads took the form of recycled connector pins which were removed from a ceramic dual in-line package (CDIP) (NTK Ceramic Co., LTD.). These connector pins provided a large, flat surface which was rigid enough to allow for wire bonding (West-Bond, 7476E) directly to the pin surface. While 4-probe electrical measurements are not sensitive to the contact electrical resistance, it is worth noting that by measuring the resistance of each pair of bonded pins the electrical resistance of an individual connection including the silver paint, CDIP pin, and wire bond was estimated to be just 5 Ω. This is small compared to the ~200 Ω measured for the gold heater film deposited on the suspended, composite samples.

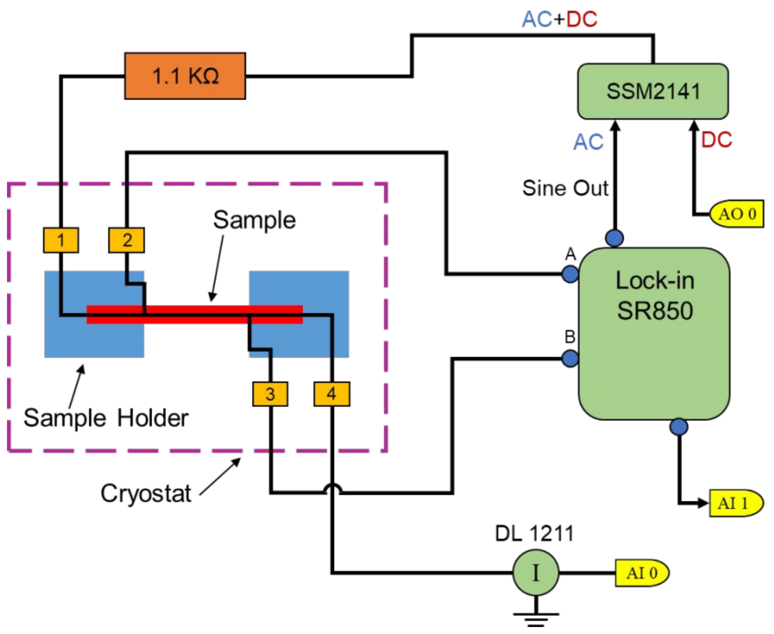

Fig. S1: Schematic representation of the steady-state DC thermal bridge measurement scheme.

A schematic diagram of the steady-state DC thermal bridge conductance measurement is given in Fig. S1. During measurement, the sample holder assembly is placed inside a cryostat (Jancis CC-450). The vacuum level of the cryostat is then lowered to be at or below 10^{-6} mbar using a turbomolecular pump (Edwards E2M1.5), and the sample holder temperature is adjusted via a temperature controller (Lakeshore 335). Here, a single lock-in amplifier (Stanford Research SR 850) is used to monitor the voltage change of the metal layer which serves as both the heater and thermometer for the thin film sample. To do so, a small sinusoidal alternating current (AC) signal, i_{ac}, is generated from the lock-in amplifier and coupled to a direct current (DC) heating source ($i_{ac} << I$) by an integrated differential amplifier (Analog Devices SSM2141). To ensure a constant current condition under each specified DC heating voltage, the coupled current sources are connected in series to a resistor (1.1 KΩ) with a magnitude significantly larger than that of the

deposited metal layer, R_0 (~200 Ω). The applied DC voltage generates Joule heat in the metal layer, and the corresponding temperature increase induces an electrical resistance change in the metal which is measured *via* the 4-point probe method by the lock-in amplifier. This change in the output AC voltages of the lock-in amplifier is monitored by a data acquisition (DAQ) board (National Instruments PCI-6052e), and the DC heating voltage is simultaneously measured by a high accuracy current preamplifier (DL Instruments Model 1211).

II. Measurement Method

By ensuring good thermal contact and minimizing radiation losses, the temperature profile of the suspended sample can be described according to:[1]

$$T(x) = T_0 + \frac{VI}{2A\kappa_s}x - \frac{VI}{2LA\kappa_s}x^2, \tag{S1}$$

where T is the temperature, V is the DC voltage, I is the DC current, A is the sample cross-sectional area, κ_s is the combined thermal conductivity of the sample and metal layer, L is the sample suspended length, and x is the distance along the suspended sample where $x(0)$ and $x(L)$ represent the contacts with the sample holder. Further, because the heating power (VI) is selected such that the temperature rise of the sample is small (< 5 K), the average temperature profile of the sample is given by:[1]

$$\Delta T = \frac{VIL}{12A\kappa_s}. \tag{S2}$$

This temperature rise of the sample will result in a corresponding increase in the resistance of the deposited gold layer such that:[1]

$$R_m = R_0 + \beta R_0 \Delta T, \tag{S3}$$

where R_m is the resistance of the metal layer after heating, R_0 is the resistance of the metal layer with no heating power applied, β is the temperature coefficient of resistance (TCR), and ΔT is the temperature rise of sample.

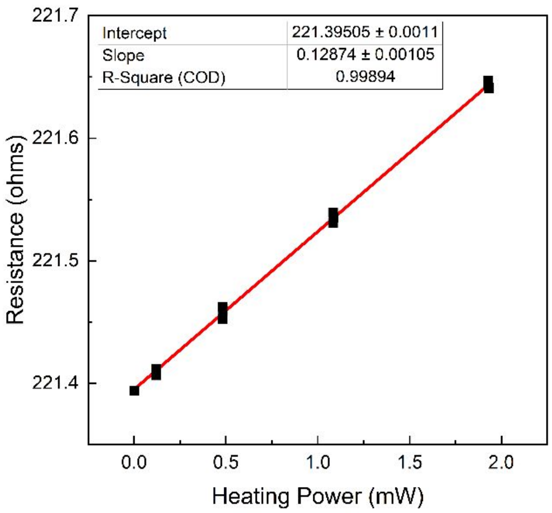

Fig. S2: Typical fitting of heater resistances for the extraction of R_0

By combining equations (S2) and (S3), a relationship can be obtained between the applied heating power and the resistance of the metal layer as described by Eqn. (S4).[1]

$$R_m = R_0 + \frac{\beta R_0 Q_h L}{12 A \kappa_s}. \tag{S4}$$

Here, the value of R_m can be obtained using Ohm's Law, and by applying a sweeping DC current at each set temperature, R_0 can be extrapolated from a linear fit of the measured resistance data (Fig. S2).

The TCR of the gold layer is then determined by calculating the slope of R_0 over the entire measured temperature range (290-310 K). For the samples in this work, the calculated TCR of the gold heater was found to have an average value of 0.00091/K at 300 K, which is in good agreement with the TCR data for 10 nm gold thin films deposited on polyimide.[2] Finally, the thermal conductivity of the samples, κ_s, including the combined thermal conductance of the layered composite, insulating PVP layer, and gold heater layer, can be determined according to Eqn. (S5).[1]

$$\kappa_s = \frac{\beta R_0 I V L}{12 A (R_m - R_0)}, \tag{S5}$$

III. Contact Thermal Resistance

In order to accurately measure the thermal conductivity of the composite samples, it is important to consider the effects of contact thermal resistance between the suspended sample and the sample holder. This contact thermal resistance can be estimated from a fin model according to:[3]

$$R_c = \frac{2}{\sqrt{h w_c \kappa_s A}\tanh\left(L_c\sqrt{\frac{h w_c}{\kappa_s A}}\right)} \tag{S6}$$

where R_c is the contact thermal resistance between the sample and sample holder, h is the contact conductance per unit area, w_c is the contact width, and L_c is the contact length. In order to ensure good thermal contact and minimize the contact thermal resistance, a commercial, electrically insulating thermal paste with a reported thermal conductivity of 15.7 W/(m·K) was applied to the copper sample holder, and the samples were pressed firmly onto the holder surface. The contact conductance per unit area, h, can be estimated from the reported thermal conductivity of the paste as $h = \left(\frac{t}{15.7}\right)^{-1}$ where t is the thickness of the contact layer. Using the plastic applicator supplied with the thermal paste, a thin layer of paste ($t < 200$ μm) could be readily achieved, and neglecting the effects of pressing the samples into the paste, the resulting value h is 78,500 W/ (m^2·K).

For the highest thermal conductivity samples, the combined thermal conductivity of the composite thin films, κ_s, and insulating PVP layer is ~20 W/(m-K), the contact width is 0.6 mm, the sample cross-sectional area is 0.038 mm^2, and the contact length is 20 mm. From Eqn. (S6), the worst-case scenario contact resistance is found to be 334 K/W, which is small compared to an

intrinsic sample resistance of 8,335 K/W. Therefore, the maximum contact thermal resistance contributes < 4% to the total resistance and is regarded as negligible.

IV. SEM Images of the Sample Cross-Sections

A representative scanning electron microscopy (SEM) image of a cross-section for each of the measured AgNW volume fractions is provided in Fig. S3.

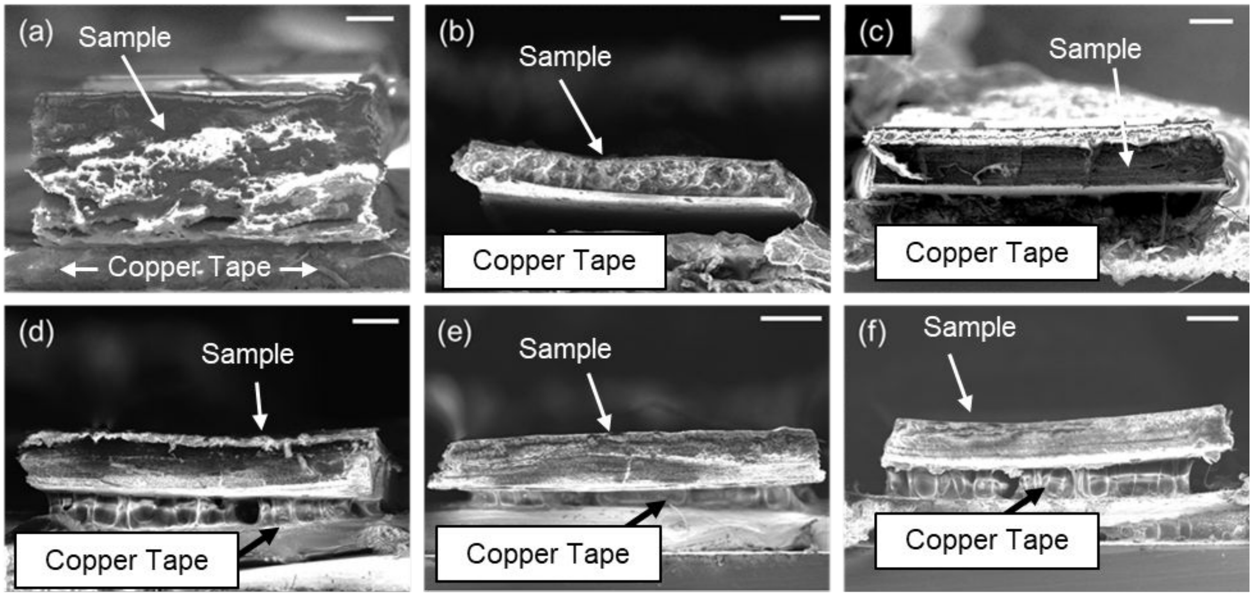

Fig. S3: SEM micrographs of the sample cross-sections for each measured AgNW volume fraction: a) 2.5%, b) 5%, c) 7.5%, d) 10%, e) 15%, f) 20%. The scale bar for each image is 100 μm.

V. Effects of Thermal Radiation

Due to the assumption of negligible radiation loss in the one-dimensional heat conduction model, an important consideration when implementing the steady-state DC thermal bridge measurement technique is to design the sample dimensions such that the radiation heat transfer from the sample surface to the surroundings is trivial as compared to the conduction heat transfer along the sample. For temperatures below ~1000 K, Cahill notes that the effects of radiation losses, Q_{rad}, can be approximated by comparing the linearized radiation coefficient, h_{rad}, to the conduction of the sample, Q_c, as given in Eqn. (S7):[4]

$$\frac{Q_{rad}}{Q_c} = \frac{h_{rad}L^2}{4\kappa_s t}, \quad \text{(S7)}$$

where $h_{rad} = 4\varepsilon\sigma T^3$, L is the sample suspended length, t is the thickness of the sample, ε is the emissivity, and σ is the Stefan-Boltzmann constant.

For the samples with a silver volume fraction of 0.025, the sample length is 6.35 mm, the sample thickness is 295 μm, and the combined thermal conductivity of the composite film and the PVP insulation layer has a measured value of 3.1 W/(m·K). Therefore, by taking the emissivity to be equal to unity at 300 K, the ratio of radiative conductance relative to the sample conductance has a maximum value of 0.067. From this the one-dimensional heat transfer model is considered valid for the PVP-AgNW composite samples.

VI. Measurement Uncertainty

Due to the relatively large size of the composite films and the corresponding high thermal conductance relative to the background, the primary source of experimental uncertainty when quantifying the thermal properties of the thin films arises from the manual placement of the electrical leads. The thermal conductivity of the measured samples is given by Eqn. (S5) and is dependent on the sample dimensions (L and A), the equipment sensitivity to the applied power (IV), and the electrical properties of the gold heater layer (β, R_0, and R_m). Following the standard error propagation analysis, the experimental uncertainty of the sample thermal conductivity, κ_s, can be calculated as:

$$\frac{\delta\kappa_s}{\kappa_s} = \sqrt{\left(\frac{\delta L}{L}\right)^2 + \left(\frac{\delta A}{A}\right)^2 + \left(\frac{\delta I}{I}\right)^2 + \left(\frac{\delta V}{V}\right)^2 + \left(\frac{\delta \beta}{\beta}\right)^2 + \left(\frac{\delta(\Delta R)}{(\Delta R)}\right)^2}, \tag{S8}$$

where ΔR represents the quantity $(R_m - R_0)/R_0$.

The copper sample holder has a machined trench with a fixed width of 6.35 mm, and as such the uncertainty of L is dependent on the sample alignment. Because samples can be adjusted after their initial placement, it is estimated that the maximum degree of misalignment is ~5° for the desired direction of perpendicular to the trench. The resulting uncertainty of $\delta L/L$ is then less than 1%. The cross-sectional areas of the composite samples were evaluated using SEM images, and repeated measurements of the area with ImageJ suggest the maximum uncertainty of the area is ~2%. This low uncertainty is due primarily to the large sample area relative to the resolution of the SEM, and, as expected, the maximum uncertainty is associated with the thinnest samples (those containing 20% volume fraction of AgNWs).

The uncertainty of I is dependent on the resolution of the measurement equipment. DC current measurements are performed with a DL instruments 1211 current preamplifier with a measurement resolution of < 1 μA at the selected instrument settings while measured DC currents are on the order of 1 mA. The uncertainty of the DC current measurement is therefore considered negligible.

During data processing, the DC voltage drop (V) across the sample is determined from the measured DC current and the resistance of the gold layer (R_m) with $V=IR_m$. Further, R_m is determined from the AC current and volage measurements such that $R_m=\upsilon_{ac}/i_{ac}$. Finally, while υ_{ac} is a known value generated from the Stanford lock-in amplifier, i_{ac} is determined from R_0 where $i_{ac}= \upsilon_{ac}/R_0$. Therefore, the uncertainty of V and R_m are dependent on the uncertainty of R_0.

R_0 values are determined from a linear regression of the measured resistance (R_m) plotted against the applied heating power as outlined in Section I. The coefficient of determination (R^2)

values for this regression operation are > 0.95 in all cases. From this R^2 value, the measurement range, and the number of data points taken for each sample at each set temperature (n = 18), it can be shown that the maximum variation of R_0 is ~0.01 Ω compared to an absolute resistance of ~200 Ω. The resulting relative uncertainty $\delta R_0/R_0$ is less than 0.01%. Following the standard uncertainty propagation, the relative uncertainties $\delta R_m/R_m$ and $\delta V/V$ can both be shown to be of similar magnitude and are therefore considered negligible.

However, while both R_0 and R_m have marginal uncertainties with regards to the resolution of the measurement equipment, the increase in the resistance of the suspended portion of the sample relative to R_0, represented by the term ΔR, is also impacted by the manual placement of the inner electrodes. Due to the good thermal contact of the sample with the copper sample holder, only the suspended portion of the samples experience a temperature increase as a result of an applied DC heating current. As a result, only the suspended portion of the sample exhibits a corresponding resistance change. Therefore, if there exists any misalignment between the inner electrodes and the edge of the machined gap in the copper holder, an error will be introduced to the quantity ΔR with magnitude $\delta L/L$ where δL is the absolute misalignment distance. The maximum observed misalignment distance was ~600 µm or 10%. The corresponding relative uncertainty $\delta\Delta R/\Delta R$ is thus also 10%.

The TCR of the gold heater, β, is determined as $\beta = (R_{02} - R_{01})/(R_{01}(T_2 - T_1))$ where R_{02} and R_{01} are the electrical resistance of the gold at set temperatures T_2 and T_1, respectively. δR_0 values have been shown to be negligible and here are unaffected by the electrode placement as only values where no heating power is applied are compared. Further, the temperature fluctuation of the cryostat has a magnitude of ~10 mK at measurement temperatures near 300 K, making the uncertainty of the set temperature also negligible. Therefore, $\delta\beta/\beta$ was taken to be negligible.

The relative uncertainty of the thermal conductivity of the composite samples, $\delta\kappa_s/\kappa_s$, calculated from Eqn. (S8) is then 10.2%.

It should be noted here that in order to account for the effects of the insulation layer the final thermal conductivity of the composite, κ_c, is calculated as $\kappa_c = \frac{\kappa_s - A_r\kappa_{PVP}}{1-A_r}$, where A_r is the area ratio of the insulation layer relative to the total sample cross-sectional area, and κ_{PVP} is the thermal conductivity of PVP. Due to the subtractive terms, the uncertainty of the numerator and denominator must be considered separately. In this way the absolute uncertainty of the numerator, denoted by the subscript n, becomes:

$$\delta\kappa_{cn} = \sqrt{(\delta\kappa_s)^2 + (\delta(A_r\kappa_{PVP}))^2}. \tag{S9}$$

For the measured thermal conductivity of the sample with an AgNW volume fraction of 0.2, $\delta\kappa_s$ has a value of 2.061 W/(m·K) (or 10.2%) compared to value of just 0.006 W/(m·K) for $\delta(A_r\kappa_{PVP})$ when assuming a 2% uncertainty on the area and using the 10% uncertainty previously determined for the PVP thermal conductivity measurements.[5] As a result, the absolute uncertainty of $\delta\kappa_{cn}$ has a value of 2.067 W/(m·K) and the relative uncertainty $\delta\kappa_{cn}/\kappa_{cn}$ is ~10.2%.

Finally, the relative uncertainty $\delta\kappa_c/\kappa_c$ can be derived as:

$$\frac{\delta\kappa_c}{\kappa_c} = \sqrt{\left(\frac{\delta\kappa_{cn}}{\kappa_{cn}}\right)^2 + \left(\frac{\delta A_r}{A_r}\right)^2}. \tag{S10}$$

Thus, the maximum uncertainty is 10.6% and was observed for the 20% AgNW composite samples. This value decreases, approaching 10.2%, as the AgNW concentration is reduced due to the increased thickness of the lower thermal conductivity samples.

VII. Modelling Uncertainty

Using a nonlinear percolation model proposed by Foygel et al.[6] the thermal conductivity of a composite can be calculated as:

$$\kappa_s - \kappa_m = \kappa_0\left(\frac{\varphi-\varphi_c}{1-\varphi_c}\right)^{\tau}, \tag{S11}$$

where κ_m is the thermal conductivity of the matrix material, κ_0 is a pre-exponential factor which depends on the thermal conductivity of individual nanofillers and the topology of the percolated network, φ is the filler volume fraction, φ_c is the critical volume fraction or the percolation threshold, and τ is an exponent that is dependent on the filler aspect ratio, p. For AgNW-PVP composites, the aspect ratio is defined as the nanowire length, l, divided by the diameter, d, ($p = l/d$). For high aspect ratio fillers ($p >> 1$), the critical volume fraction is related to the aspect ratio such that $\varphi_c \approx 0.6/p$.[6] For the AgNWs used in this study, the aspect ratio is 571, and the critical volume fraction is 0.001. The remaining unknowns in Eqn. (S11) are κ_0 and τ, which are taken as fitting parameters.

The fitting parameters κ_0 and τ are found by rearranging Eqn. (S11) as:

$$\log\left(\frac{\varphi-\varphi_c}{1-\varphi_c}\right)\tau + \log(\kappa_0) = \log(\kappa_s - \kappa_m), \tag{S12}$$

and using a simple linear regression model to fit the experimental data. The reported uncertainties for κ_0 and τ, are representative of the 95% confidence interval values of the slope and y-intercept using the standard approach for regression uncertainty.

References:

(1) Zhang, Q.G.; Cao, B.Y.; Zhang, X.; Fujii, M.; Takahashi, K. Size effects on the thermal conductivity of polycrystalline platinum nanofilms. *J. Phys: Condens. Matter*. **2006**, 18, 7937.

(2) Oliva, A.I.; Lugo, J.M.; Gurubel-Gonzalez, R.A.; Centeno, R.J.; Corona, J.E.; Avilés, F. Temperature coefficient of resistance and thermal expansion coefficient of 10-nm thick gold films. *Thin Solid Films*. **2017**, 623, 84-89.

(3) Bifano, M.F.; Park, J.; Kaul, P.B.; Roy, A.K.; Prakash, V. Effects of Heat Treatment and Contact Resistance on the Thermal Conductivity of Individual Multiwalled Carbon Nanotubes Using a Wollaston Wire Thermal Probe. *J. App. Phys*. **2012**, 111, 054321.

(4) Cahill, D.G. Thermal conductivity measurement from 30 to 750 K: the 3ω method. *Rev. Sci. Instrum*. **1990**, 61, 802-808.

(5) Fitzgerald, M.L.; Zhao, Y.; Pan, Z.; Yang, L.; Lin, S.; Sauti, G.; Li, D. Contact Thermal Resistance between Silver Nanowires with Poly (vinylpyrrolidone) Interlayers. *Nano Lett*. **2021**, 21, 4388-4393.

(6) Foygel, M.; Morris, R.D.; Anez, D.; French, S.; Sobolev, V.L. Theoretical and computational studies of carbon nanotube composites and suspensions: Electrical and thermal conductivity. *Phys. Rev. B*. **2005**, 71, 104201.
